# A Plea for Conservative Oral Surgery with Practical Illustrations

**Published:** 1897-03

**Authors:** G. Lenox Curtis

**Affiliations:** New York City.


THE
AMERICAN JOURNAL
—OF—
DENTAL SCIENCE.

VOL. XXX. BALTIMORE, MARCH, 1897. NO. 11.

ARTICLE I.

### A PLEA FOR CONSERVATIVE ORAL SURGERY, WITH PRACTICAL ILLUSTRATIONS.

BY G. LENOX CURTIS, M. D., NEW YORK CITY.

Read before the Atlanta Meeting of the American Medical Association.

There is, perhaps, no other department of surgical practice in which the general surgeon, trained in the medical schools alone, is so deficient as in oral surgery. He clings to the ways of the ancients, and makes no effort to improve his methods in oral and facial surgery. The fault is not so much his as it is that of the system under which he was educated. For, notwithstanding all that has been demonstrated by Profs. Garretson and Tomes, the medical colleges persist in declining to annex to their curricula the special line of work regarding the facial region which would seem to be of paramount importance, in view of the æsthetic factor involved.

The medical student of to-day receives no training in oral and facial surgery; so that the general surgeon may be excused for not practising that which he has not been taught. Even our modern text-books contain many of the identical illustrations and much of the advice upon this topic which were published in the "forties." The surgeon trained under such auspices must, in order to advance in oral surgery, create, by his own observation and skill, better methods. To such a one, the Langenbeck operation, the opening through the face for the resection of the jaw, for the removal of tumors and necrosis, trephining below the eye to gain access to the antrum of Highmore, the resection of nerves by cutting through the face, may seem justifiable. But to the man who has seen such operations performed through the oral cavity, so that no visible external scar is left, such practice seems like butchery, and the practitioner who still persists in the old way is almost guilty of malpractice.

That the condition of oral surgery as practised by the average general surgeon is entirely because of the lack of better teaching in the schools, and that he will accept better methods when their value is demonstrated to him, is evidenced by personal experience. Just prior to the writer's appointment on the staff of the New York Post-Graduate Medical School, every general surgeon of the faculty who had a vote cast it against him, and he was informed that it was because they did not wish to see this specialty established. It was not long, however, before some of these, recognizing the beneficence of the conservative method, applied for instruction and were frequently found at his clinic.

The late Prof. Garretson met with a similar, though more resisting, opposition twenty-five years ago, in consequence of which he was forced to join with a dental college, when the work that he did, great as it was, fell short of what it would have accomplished had he been connected with a medical school.

Why the faculties of the medical institutions persist in ignoring the advances which have been made in oral surgery, which it would seem have reached a point to demand their incorporation into the medical curriculum, is past comprehension. In view of the facts, one might almost question whether it is due to selfishness, self-sufficiency, or politics that this field is so entirely neglected. Certainly the present course is not in the line of scientific advancement.

It seems now time that America, if she wishes to lead in medicine, as in many other professions, should establish a medical institution devoted to the higher education of students in the department of oral surgery and other neglected subjects, such as nervous diseases, rheumatism, gout, and the treatment of the kidneys, and thus give free and unencumbered scope to the inquiring mind willing to devote itself to this work, and give the world the benefit of the results of its investigations.

To illustrate the need of a better knowledge of oral surgery among general surgeons, allow me to quote the following cases from practice:

April 19th, 1893, Mrs. M., about thirty-five years of age, was brought to me by her dentist, giving the following history: For several years she had had trouble with her teeth, some of them being abscessed, the trouble coming and going from time to time. About February 18th, the left side of her face became swollen, and a severe pain was left in the jaw, the swelling gradually extending to the temporal region. A week afterwards the presence of pus was detected. In the meantime her physician applied alternately cold and hot applications, principally poultices, which resulted in the discharge of pus into the mouth. Three weeks later the face was still swollen and hard, and the jaws were closed. The temporal abscess was aspirated, and the pus drawn off; but as the difficulty showed no abatement, the patient was brought to the city for treatment. My examination showed the cheek slightly swollen, with considerable swelling in the temporal region. The deep fluctuation showed the formation of pus under the temporal muscle. There was a hardened lump of the size of a peanut near Stenon's duct, and the jaws were almost closed and rigid. The inferior left bicuspid, which had been abscessed and troublesome for many years, had been extracted some two months previously, but the socket had refused to heal; there was also periosteal and sub-periosteal inflammation throughout the entire labial and buccal surface of the inferior maxilla on the left side extending from the central incisor back to and up along the ramus of the jaw. From the inflammatory centre, in my opinion, both the temporal abscess and the one in the cheek had formed, and I demonstrated it to the dentist as the cause.

June 1st, the patient again presented herself at my office with the following additional history, begging me to operate for her: She had been advised to go to a general surgeon whom she was assured was a specialist in oral surgery, in fact a specialist in every branch of surgery. He had performed six torturing operations in six weeks without satisfactory results; and stated as an excuse for the seventh operation, which he proposed doing, that he had not known and did not know the cause of her trouble, and that he would make an incision from the temporal region to the lower portion of the cheek, a distance of about six inches, opening up the face to the bone to ascertain where the cause lay. This she refused to submit to and left the hospital.

Examination revealed the following condition: The patient showed a great loss of flesh; was feeble, anæmic and feverish, tongue badly coated, bowels constipated; she had been obliged to submit to the loss of her hair to facilitate the dressing of the wounds. The jaws were rigidly set, and the patient swallowed even liquid with great difficulty. The face was badly swollen and indurated, pitted on pressure, and bore a strong resemblance to liver.

An abscess which pointed in the cheek near the angle of the mouth was almost ready to break through the skin. There was also a deep red spot under the left eye accompanied by a puffy condition with fluctuation, such as one often observes in antral disease, another of similar nature, about an inch in circumference, was situated at the external angle of the eye. There was an ugly suppurating, granulating wound immediately anterior to the ear, and extending from the middle half to an inch above it, gaping open for an inch, from which pus flowed freely. Protruding from this was a drainage tube, which passed down through the wound and opened into the mouth immediately below Stenon's duct.

The zygoma was separated from the malar bone by necrosis, its periosteum was denuded along the entire posterior surface, and the bone also necrosed. While the disease had become greatly aggravated since my first examination, and the patient's health had been much impaired, the most unfortunate complication was facial paralysis confined to this side, This the patient said had followed one of the operations at the hospital.

Realizing that there was no time to lose, we concluded to operate at once. Under ether, an opening was made through the mucous membrane into the cheek abscess immediately below Stenon's duct, near where the drainage tube entered the mouth, and several ounces of pus were evacuated.

The granulations and sac were curetted away, leaving only the skin unbroken. The wound was antiseptically packed. An incision was made through the gum and periosteum extending from the cuspid back to and along the ramus of the jaw. This was found full of pus and granulating tissue which extended to the top of the coronoid process, beyond which I could readily pass a probe up to and under the aponeurosis of the temporal muscle. Granulations and debris were also thoroughly curetted away and the wound packed. A similar condition existed under the temporal muscle which was treated in the same manner. Several ounces of pus and debris were removed. The wound which was made at the hospital was treated in like manner. The necrosed bone along the lower border of the zygomatic arch, and the malar bone which had become separated as above noted, was likewise removed. The necrosis here was quite extensive, and extended over the entire tuberosity of the superior maxillary. The inflamed places under and at the angle of the eye were not opened into at this time, as we hoped that as these greater wounds healed, the minor troubles would also disappear. The wounds were dressed twice daily for a week, during which time large quantities of pus continued to flow until the industrial condition disappeared. As this diminished the wounds were dressed daily. The temporal wound was the slowest to heal. Finding the inflammation under and at the angle of the eye showed little signs of abating, although cold compresses were applied constantly, I concluded to open and remove the cause.

On June 6th, by use of cocaine to relieve pain, I passed a knife through the mucous membrane just above the left superior second bicuspid, and by means of a grooved director, dissected away the tissues until the abscess at the angle of the eye was reached. I then made an incision in the periosteum one-half inch in length, through which I was able to curette and remove fully two drachms of pus and several flakes of dead bone. This wound was treated in a similar manner to the others, and readily healed. The abscess immediately below and near the internal angle of the eye was treated in a like manner, and with like results, the opening through the mucous membrane being made on a line with the lateral incisor. All wounds were healed within two weeks, and the swellings and the induration of the face entirely disappeared. The ugly scar in the temporal region was then dissected out, and the parts were drawn together by sutures and adhesive plasters, until healed, leaving only a slight linear scar.

The patient was dismissed and returned to her home, June 22, with all the wounds healed, the complete use of her jaws and the appearance of her face returned to its normal condition, save the marked paralysis which resulted from the treatment between April 19th and June 1st. Before leaving the city she presented herself at the office of the surgeon who did these first operations, and showed him the results of conservative oral surgery, asking him to note well the facial paralysis which he admitted to her he was the cause of.

Loyal to my fellow practitioner I shielded him from his error, and prevented suit being brought for malpractice by her husband against this surgeon, who claimed to be a specialist in everything, by stubbornly declaring that I would be a witness for the defendant and swear that in my judgment he treated the case as taught in our college and text-books and according to his best ability.

To impress more definitely upon the minds of the readers of this paper perhaps the most potent cause of temporal abscess, I will narrate the history of another and similar case to the one already given.

Mr. L. presented himself with the characteristic swell-in the temporal region and complaining of great pain. Deep fluctuation was readily observed, denoting the presence of pus beneath the temporal muscle. The gums along the alveolar border extending back of the cuspid were highly inflamed ond œdematous. The root of the first bicuspid tooth was found almost covered by the gum and abscessed. This had from time to time given him considerable trouble. Attributing to this the cause of the trouble, I removed it and found that I could pass a probe beneath the periosteum as far back as the wisdom tooth. An incision was made through the gum and periosteum extending well back along the ramus. This enabled me to pass a large probe beneath the periosteum up the ramus and beyond the coronoid process, following the temporal muscle until I had reached the abscess, the pus from which flowed freely down beside the probe and out into the mouth. The bone immediately under the periosteum was covered with granulations and pus. This, along with that underlying the temporal muscle, was curetted away. The wound in the jaw was packed, while that in the temporal region was dauched and sterilized twice daily, and applications of ice were made to the exterior. The stiffness of the jaw at once began to improve, and in a few days it was normal in its action. The treatment covered a period of ten days when the patient was dismissed cured, and now nearly four years have elapsed without any sign of return.

The origin and progress of this case were identical with those of Mrs. M. already mentioned, and had she had similar treatment at the same stage of her disease, the result would have been as happy as in this case and without any external disfigurement.

A similar but more perplexing condition than that referred to above, is one with the following history and results: Four years prior to May, 1892, the patient, while suffering from a severe pulpitis caused by exposure of the pulp in the inferior left third molar, had the tooth extracted, which was immediately followed by excruciating pain, but of a vastly different character from that which he had previously suffered He likened the pain unto a severe bruise. The pain continued to increase, and the following day he returned to the dentist and insisted upon his extracting the second molar, although it was not decayed. This the dentist did reluctantly, thinking that perhaps the extraction of the wisdom tooth might have ruptured the nerve, because of the fact that the ends of the roots were bent like a hook. The pain continued for several days, when another dentist was consulted, who continued the process of extracting teeth, but with no relief.

Medical counsel was then sought, but the case baffled all treatment for several months. The patient's health diminished, and the pain continuing in the jaw, he sought relief at the hands of a third dentist, who, like a true knight of the forceps, removed the remaining teeth of both left superior and inferior jaws. The shock to the nervous system and the profuse hemorrhage which followed, owing to the weakened physical condition of the patient, gave him temporary relief. But the old trouble soon returned, and he found himself back under medical treatment—from which he realized no improvement, finally abandoning his business and becoming an invalid.

After the lapse of two years, he sought the aid of a general surgeon, who, concluding that the trouble was in the gums and alveolar process in the inferior maxilla, cut and chiselled them entirely away, but to no avail. For two years more suffering and medical treatment continued until the patient was little short of a wreck and all but insane. He had lost forty six pounds in weight, was emaciated and anæmic, and he grew despondent and longed for death to relieve him of his agony.

Diagnosis of the seat of the trouble was based upon the early history of the case at the time of the extraction of the wisdom tooth. It was plain that the inferior dental nerve had been lacerated in the localty of the wisdom tooth, and that no relief could be hoped for until the nerve was severed between it and its centre. To make sure of the result, I decided to remove the entire nerve within the jaw. An incision, about an inch long, was made through the mucous membrane, directly above and back of the location of the wisdom tooth, and the tissues were separated until the nerve was reached as it entered the inferior dental foramen. The nerve was caught up and held with the bull-dog forceps, and served at this point.

An incision was then made over the mental foramen, the tissues dissected away, and the dental nerve, where it emerged, was separated. The forceps was then tightly grasped and with a steady tension the nerve was drawn out of the canal its entire length. The hemorrhage was readily controlled by means of hot water, but owing to the general flabby condition of the tissues, and to the fear of a secondary hæmorrhage, the wound was packed and allowed to fill in by granulation. The patient soon recovered from the ether, declaring on his return to conciousness that "for the first time in years" he "was free from pain." The parts healed rapidly and no untoward symptoms followed the operation, save a little numbness noticeable at times in the left half of the lower lip. The patient soon recovered health, strength and weight, returning to business in two months, and there has been no return of the trouble.

I will next report a very remarkable case which came to me in April, 1892, as it may materially assist in the treatment of orchitis—being the details of one of several cases coming under my observation.

Mr. B., aged thirty-seven years, with no specific history, was referred to me by Dr. L. Bolton Bangs, of New York City, to whom the patient had been brought for consultation, with the request that I examine his jaw to ascertain whether there was any oral lesion to account for a pain complained of that day. On inquiring into the dental treatment received by Mrs. B., I drew from him the following statement: Five years before the gums over the inferior left wisdom tooth, which was retarded in its eruption, became suddenly swollen and very painful. He applied to his dentist, who, in attempting to extract the tooth, broke off the crown, leaving the root, over which the gum healed, completely embedding it. Since that time he had realized uncomfortable sensations on that side of the face with some soreness of the inferior second molar which baffled the skill of those hunting for the cause.

The patient supposed the root to have been extracted at the time the crown was separated from it. Three years following this visit to the dentist, he was attacked by excruciating neuralgic pains in the left side of the face, which, until two months before calling on me, unfitted him for business. This pain gradually worked its way down the left side of the body, extending to the groin and left testicle, which became inflamed, swollen and troublesome. All efforts on the part of his surgeon to relieve his suffering were unavailing.

One of the peculiar features of the treatment in this case is, that when hot or cold applications were made to the testicle, the pain ceased in it, but immediately appeared in the left side of the face. As soon as the applications were removed, the pain returned to the testicle.

After examining him, I concluded it was a case of metastasis, such as is frequently connected with mumps. I sent the patient back to Dr. Bangs with the following note: "I believe I have found the cause of this long and persistent neuralgia, and that, if I operate the patient will no longer have need of your services, as the orchitis will disappear with the healing of the wound."

The patient was not long away, for the wide awake specialist sent him back with a note stating, "You cannot operate too quickly to suit me, you have awakened my curiosity. I am interested and will be pleased to follow the case with you."

Examination revealed a slight necrosis of the alveolar process immediately back of the inferior second molar, the pulp in the distal root of which was dead and abscessed, the pulp in the anterior root being vital and exposed. There was a large cavity in the distal surface of the tooth, below the enamel, concealed by the gum, hence the long continued soreness of the tooth. I extracted this tooth and removed the slight diseased condition made by the abscess.

The cavity in my opinion was caused by secretions forming between the second molar and the wisdom tooth, which abutted horzontally against it. There was no satisfactory evidence of the extraction of the root of the wisdom tooth, and the gum over it appeared normal. But to be sure that all possible cause for the pain was removed, I laid open the gum, cut through the periosteum and bone, and suddenly struck upon the root which had so long been buried. In examining to ascertain its position in the jaw, I plunged my probe into the living pulp of the root. You can better imagine the result of this thrust than I can here tell it. The patient's actions reminded me of the antics of a jumping jack when the string is pulled. Under an anæsthetic I dissected out and removed this root. The wound healed readily, all pain ceased with the operation and the patient made a complete recovery. There was no swelling of the testicle the day following the operation—all pains and soreness disappearing within forty-eight hours. It has not since returned, the patient being restored to perfect health.

It gives me great pleasure to mention here the praiseworthy attitude of Dr. Bangs in contra-distinction to that of the surgeon who handled the case of Mrs. M.

The next case is one in which the cause is so plainly discernible that it is liable to be overlooked. An old lady in her "sixties" had for many years suffered intensely from facial neuralgia. After repeated failures of medical skill, the patient was transferred to the general surgeon, who, in six years did several operations in the right superior and inferior maxillæ, resecting the nerves and deforming and disfiguring that side of the face. When I was called to see the patient all the teeth had been extracted from the right side of the mouth, but the pain remained incessant—less, however, at night, and when she lay upon her right side, or when her face was swathed in flannel and protected from cold blasts of air.

Her general health in consequence of her long continued suffering was greatly enfeebled. Examinations of the mouth was unsatisfactory, so I looked further for the cause, which I apparently found in two large "seed" moles, one of which was situated immediately in front of the ear, and the other three inches below on the affected side of the face. A few drops of cocaine were injected beneath the tissues at the base of these moles, and the skin dissected sufficiently for a small ligature to be thrown about them and firmly tied. The moles were snipped off with scissors and the stumps cauterized with nitrate of silver. The ligatures came away with the sloughs which formed, and the wounds healed without further treatment.

An examination of the moles revealed an exposure of the nerves, which were also intensely inflamed.

Several months subsequently I received a letter from the patient's son, stating, "Since the simple operation which you did for my mother, she has not experienced the slightest pain and daily blesses you."

Similar cases are common where patients have travelled nearly the world over consulting physicians in search of relief at an enormous expenditure of time and money.

The operations described are not new to those familiar with the progress of oral surgery as worked out by the more advanced members of the dental profession, but the fact remains that the general information given to the medical student is insufficient for the proper handling of these cases.

I speak within bounds when I say that maltreatment at the hands of men ignorant of the higher development of this branch of surgery has given me the greater number of my patients.

There is no question that the more cultivated dentists know the surgery of the mouth better than the surgeon who has been only generally trained; know better also the relations of disorders of the oral cavity with contiguous and distant tracts, and are better prepared to diagnose the cause of many obscure lesions connected with those relations.

I would therefore recommend to the surgical profession, particularly to those who have had no special opportunities for studying the diseases of the mouth, the calling in of a skilful dentist, preferably one who has been medically educated, at least for the benefit of his judgment in diagnosis, whenever there is room to suspect oral complications.

Our medical schools will not do their entire duty by their students until they add to their list of teachers dentists of the ability to instruct their students in diseases following affections of the teeth; and our text-books will be lacking until they give proper attention to oral surgery as viewed from a conservative standpoint.—*N. Y. Medical Journal.*

